# The Extent of Alcohol-Related Problems Among College and University Students in Norway Prior to and During the COVID-19 Pandemic

**DOI:** 10.3389/fpubh.2022.876841

**Published:** 2022-05-26

**Authors:** Ove Heradstveit, Børge Sivertsen, Kari-Jussie Lønning, Jens Christoffer Skogen

**Affiliations:** ^1^Regional Centre for Child and Youth Mental Health and Child Welfare, Norwegian Research Centre, Bergen, Norway; ^2^Centre for Alcohol and Drug Research, Stavanger University Hospital, Stavanger, Norway; ^3^Department of Health Promotion, Norwegian Institute of Public Health, Bergen, Norway; ^4^Department of Research & Innovation, Helse Fonna HF, Haugesund, Norway; ^5^Department of Mental Health, Norwegian University of Science and Technology, Trondheim, Norway; ^6^The Norwegian Medical Association, Oslo, Norway; ^7^The Student Welfare Association of Oslo and Akershus (SiO), Oslo, Norway; ^8^Centre for Evaluation of Public Health Measures, Norwegian Institute of Public Health, Oslo, Norway

**Keywords:** alcohol use, alcohol-related problems, Alcohol Use Disorders Identification Test, college students, university students, COVID-19

## Abstract

**Aim:**

To provide estimates of the distribution of alcohol-related problems in a national sample of college and university students in 2021, i.e., during the COVID-19 pandemic, in comparison with pre-pandemic data from 2018.

**Design:**

Longitudinal data from linkage of two recent national health surveys from 2018 to 2021.

**Setting:**

Students in higher education in Norway (the SHoT-study).

**Participants:**

8,287 fulltime students (72.5% women, 27.6% men) that were 18 years or more at the time of the first survey in 2018, and 21 years or more at the time of the second survey in 2021.

**Measurements:**

The Alcohol Use Disorders Identification Test (AUDIT) was used to assess potential alcohol-related problems.

**Findings:**

37.0% of male students and 24.1% of female students reported either risky, harmful, or dependent alcohol use in 2021, compared with 55.0% of male students and 43.6% of female students in 2018. This decrease in alcohol-related problems was most pronounced for dependent alcohol use, where we observed a 57% relative reduction among male students (from 3.5% in 2018 to 1.5% in 2021) and a 64% relative reduction among female students (from 1.4% in 2018 to 0.5% in 2021).

**Conclusions:**

The present study demonstrated a sharp decline in alcohol-related problems among students during the COVID-19 pandemic, that were present across gender, age groups, and geographical study locations. Universal preventive measures to limit students' alcohol use should be considered when restrictions related to the pandemic is lifted.

## Introduction

It is well-documented that alcohol-related problems are frequent among students in higher education (1–3). Despite the facilitating role of alcohol use in many student social settings (4), alcohol use is associated with a range of negative outcomes (4, 5). An alarmingly high proportion of students in higher education display alcohol use habits that classify as potentially harmful, with prevalence estimates ranging from 21 to 82% (3, 6–9). Recent estimates from Norway showed that more than half of male students and 4 out of 10 female students reported risky, harmful, or dependent alcohol use, as measured with the Alcohol Use Disorders Identification Test (AUDIT) (2).

Monitoring trends in alcohol use among students in higher education is important for several reasons. It provides a way to assess the extent of the problem over time, which may shed light on how specific preventive measures, national policies, and other factors may have affected alcohol use in student settings [e.g., (10, 11)]. One such factor became evident during 2020 and onwards, namely the COVID-19 pandemic. In Norway, the 12th of March 2020 marked the start of a lockdown of the society, with a range of measures aimed at social distancing. For students in higher education this meant that all teaching that had previously taken place at college- or university campuses, was delivered digitally with the students residing in their respective homes. The nightlife was practically closed, and restrictions on social contact dramatically reduced the possibilities of meeting other students in social settings.

A recent study from 21 European countries, including over 30,000 adults, reported that alcohol consumption declined in all the European countries except Ireland and the United Kingdom during the first months of the pandemic (12). On the other hand, a review and meta-analysis of 128 studies from 58 countries, that included data from both general populations and specific groups (e.g., students in higher education), revealed that the overall mean change in alcohol consumption was non-significant from prior to after onset of the COVID-19 pandemic (13). Out of the included studies in the review, some studies reported statistically significant increases and others significant decreases in alcohol use, and contextual factors were highlighted as important to assess when trends in alcohol use are investigated.

Due to the particularly high alcohol use prior to the COVID-19 pandemic among students in higher education (3, 6–9), which to a large degree is related to social contexts (14), it could be argued that it is likely that this group could see a reduction in alcohol use during times when social contact is restricted. Consistent with this interpretation, most international studies on students in higher education report a decrease in alcohol use during the pandemic (14–18). It is, however, important to investigate this hypothesis empirically, as some reports suggested that—at least very early in the pandemic—increases in student alcohol use were observed (19, 20). Moreover, it could also be argued that these high initial levels of pre-pandemic alcohol consumption among Norwegian students would indicate a potential increase in alcohol use during the pandemic. This hypothesis is actualized by a recent European study which reported that changes in alcohol consumption during the first months of the COVID-19 pandemic were unevenly distributed within the population. Among people that had an initial alcohol consumption within the upper decile of the population, alcohol use actually increased, in contrast with a declining consumption in other groups of the sample (21).

The present study is the first to investigate alcohol use among Norwegian students in higher education in the second year of COVID/2021. We employ a unique linkage of two cross-sectional surveys with comparable data on student's alcohol use, highlighting contextual factors such as age, gender, and geographical locations. Consistent with previous studies from student populations, we hypothesized that alcohol use and alcohol-related problems would decrease from 2018 to 2021 among Norwegian higher education students, as social distancing measures have curbed students' opportunities of socializing to a large degree.

## Materials and Methods

### Procedure and Setting

The SHoT study (Students' Health and Wellbeing Study*)* is a national survey of students in higher education in Norway. The SHoT study was initiated by the three largest welfare organizations in Norway and was conducted as a joint effort between these organizations and the Norwegian Institute of Public Health (NIPH). To date, four cross-sectional surveys of the Norwegian student population have been completed (2010, 2014, 2018, and 2021), and all four waves were collected electronically through a web-based platform. All parts of the SHoT study, including the planning of research questions, selection of study questionnaires, piloting, collection of data, as well as utilization of data and findings, were conducted in close collaboration with the student welfare organizations in Norway, where student representatives were present. Only students with Norwegian citizenship were included in the study.

The *SHoT2018* survey has been well-described elsewhere (9). In short, it consisted of survey responses from 50 to 054 students that completed the online questionnaires, providing a total response rate of 30.8%. The *SHoT2021* survey was a follow-up survey to address student health and wellbeing during the COVID-19 pandemic and was conducted between March 1 and April 6, 2021. This was a shorter health survey focusing specifically on mental health outcomes during the COVID19-lockdown. In all, 181,828 students were invited to participate, of which 62,498 students completed the survey, yielding a response rate of 34.4%. During the time of the data collection there were several national and regional restrictions. Examples included banning of all organized indoor sporting and recreational activities for adults, all campuses were closed to students in higher education, and all teaching activities were to be carried out digitally. For more details about restrictions during the collection of SHoT2021 [see (22)].

Although all SHoT studies are conducted separately as cross-sectional studies, for purposes of the present study, we used a nested longitudinal dataset comprised of individuals who participated both in 2018 and 2021 (*n* = 8,684).

### Ethics

The Regional Committee for Medical and Health Research Ethics in Western Norway approved the SHoT2018 study (no. 2017/1176) and the SHoT2021 study (no. 176205). Informed consent was obtained electronically after the participants had received a detailed introduction to the study.

### Measures

#### Demographic Information

All participants were asked to indicate their gender and age. Data on gender was available for 8,679 individuals (99.9% of the sample), and the categories included “male,” “female,” and “other” (*n* = 40, 0.5%). Thus, the eligible sample included 8,639 individuals (99.5% of the sample), of which 2,380 (27.6%) were male students and 6,259 (72.4%) were female students. We used the variable on age from the SHoT 2021 survey (spanning from 20 to 52 years, median = 24 years, interquartile range: 23–26 years), and valid data was available on 8,521 individuals (99.5% of the sample). The participants were assigned to four age groups: 21–22 years, 23–25 years, 26–28 years, and 29+ years.

#### Geographical Study Location

Geographical area of the study location was available for 8,419 individuals (96.9% of the sample). These areas included: South-Eastern Norway, Western Norway, Central Norway, and Northern Norway.

#### Alcohol-Related Problems

The Alcohol Use Disorders Identification Test (AUDIT) is a widely used instrument developed by the World Health Organization for identifying risky or harmful alcohol use (23, 24), and was used to identify potential alcohol-related problems. AUDIT includes 10 items that measure the frequency, typical amount and episodic heavy drinking frequency (items 1–3), alcohol dependence (items 4–6) and problems related to alcohol consumption (items 7–10) (25). The AUDIT scores range from 0 to 40. In the original report from Babor et al. the following cut-offs for AUDIT scores were proposed: 0–7 (No or low risk), 8–15 (risky alcohol use), 16–19 (harmful alcohol use), and 20–40 (dependent alcohol use) (23, 24). Abstainers, as indicated by a score = 0 on the AUDIT questionnaire, were thus assigned to the group “No or low risk.” We used these ranges in accordance with a recent previous publication from Norwegian students in higher education (9). In our sample, 8,637 individuals (99.5% of the sample) had valid scores on the AUDIT questionnaire in both 2018 and 2021.

### Analytical Sample and Statistics

The participants that lacked information about age, gender, study location and AUDIT-scores were excluded from the analytical sample, yielding a final sample size of 8,287 (95.9% of the eligible sample).

We first summarized the descriptive characteristics of the sample, in terms of gender, age, and geographical study location of all participants. Secondly, we described time-trends in frequency of alcohol consumption (AUDIT-item 1), usual consumption (AUDIT-item 2), and binge drinking (AUDIT-item 3) in the student population across the years 2018 and 2021. These analyses were also stratified by gender. Thirdly, we analyzed time-trends in the distribution of normal, risky, harmful, and dependent alcohol use, as well as mean AUDIT scores, from 2018 and 2021. These analyses were conducted separately by groups (i.e., gender, age groups, and geographical study locations). All proportions were calculated using a multinomial logistic regression model for associations between the groups and levels of alcohol-related problems, after which the “margins” command in STATA was used to calculate rates of alcohol-related problems per group (26). This method was conducted separately for each survey year, and included adjustments for relevant control variables (i.e., rates of alcohol use stratified for gender were adjusted for age and geographical location, rates of alcohol use stratified for age group were adjusted for gender and geographical location, and rates of alcohol use stratified for geographical location were adjusted for gender and age). We were thus able to establish adjusted rates of alcohol-related problems with 95% confidence intervals. For each gender, age group, and geographical study location we used Wilcoxon signed-rank tests to assess the statistical significance of the overall change in alcohol-related problems from 2018 to 2021. Changes in proportions of risky, harmful, and dependent alcohol consumption from 2018 to 2021 was estimated using McNemar's tests separately for each of the three risk levels when compared to the remainder of the sample. The binomial (exact) values were used to ascertain statistical significance to account for the relatively low number of discordant pairs (i.e., individuals with a change in risk category from 2018 to 2021). We also generated an alluvial plot to identify the changes in AUDIT-categories between 2018 and 2021. Due to the small number of participants in dependent category, the dependent and the harmful category was combined in the alluvial plot. STATA version 16 was used for all analyses (27), except for the alluvial plot which was generated using RStudio (28, 29) and the package “easyalluvial” (30).

## Results

### Descriptive Characteristics

Descriptive analyses of the sample showed that the sample consisted of most females (73%) and that the most prevalent age group was 23–25 years (64%, Table 1). Participants from all geographical study locations were represented, but a considerable proportion of the students had their study location in Southeastern Norway (46%). Compared to the SHoT2018 sample, participants responding to both surveys were more likely to be female (72.6% vs.69.1%), younger (21.7 years vs.23.2 years), and to have higher AUDIT score (7.5 vs.7.2, all *ps* < 0.001).

**Table 1:** Descriptive characteristics of the sample (*n* = 8,287).

	**Total %(*n*)**	**Females %(*n*)**	**Males %(*n*)**
Gender	100 (8,287)	72.5 (6,004)	27.6 (2,283)
Age groups			
21–22	10.4 (860)	11.0 (657)	8.9 (203)
23–25	63.7 (5,278)	64.6 (3,880)	61.2 (1,398)
26–28	19.8 (1,637)	18.9 (1,132)	22.1 (505)
29+	6.2 (512)	5.6 (335)	7.8 (177)
Geographical study location			
Southeastern Norway	45.5 (3,773)	46.8 (2,810)	42.2 (963)
Western Norway	22.5 (1,866)	22.7 (1,364)	22.0 (502)
Central Norway	24.6 (2,035)	23.2 (1,391)	28.2 (644)
Northern Norway	7.4 (613)	7.3 (439)	7.6 (174)

### Changes Related to Gender

**Figure 1:**
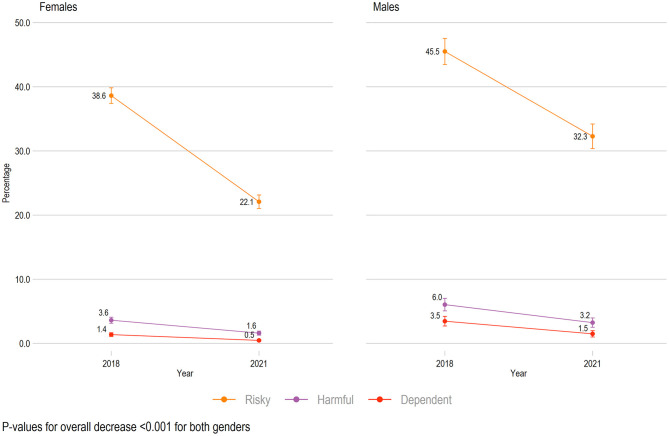
Trends in alcohol use across genders from 2018 to 2021 (*n* = 8,287). All rates are adjusted by age and geographical study locations. Scores are based on Alcohol Use Disorder Identification Test: Risky = range 8–15, Harmful = range 15–19, Dependent = range 20–40. Rates of “No or low risk alcohol use” are not shown.

There was a decrease in frequency (AUDIT-item 1), usual consumption (AUDIT-item 2), and binge drinking (AUDIT-item 3) from 2018 to 2021 for both females and males (Table 2), across age groups, and across geographical study locations (all *ps*< 0.001). There was also a significant decrease in the mean alcohol problems scores (total AUDIT score) for females from 2018 (*M* = 7.2, SD = 4.5) to 2021 (*M* = 5.3, SD = 3.9), *p* < 0.001, and for males from 2018 (*M* = 8.5, SD = 5.2) to 2021 (*M* = 6.7, SD = 4.6), *p* < 0.001 (Supplementary Table A1). The proportion of individuals with no or low risk alcohol use had a relative increase of 35% for females (from 56.4% in 2018 to 75.9% in 2021), and 40% for males (from 45.0% in 2018 to 63.0% in 2021), during the same period. For both genders, a significant decrease was observed in both risky, harmful, and dependent alcohol use (all *p*-values < 0.001, Figure 1). There was a 57% relative decrease in the proportion of dependent alcohol use for male students (from 3.5% in 2018 to 1.5% in 2021) and the corresponding number was 64% for female students (from 1.4% in 2018 to 0.5% in 2021).

**Table 2:** Distributions of (a) frequency of alcohol consumption (AUDIT-item 1), (b) usual consumption (AUDIT-item 2) and (c) binge drinking (AUDIT-item 3) from 2018 to 2021.

	**2018**	**2021**	***P*** **-value**		**2018**	**2021**	***P*** **-value**		**2018**	**2021**	***P*** **-value**
**Total**											
Frequency of alcohol consumption			<0.001	Usual consumption^a^			<0.001	Binge drinking (6 units or more)			<0.001
Never	7.3%	9.4%		1–2	12.1%	33.6%		Never	9.7%	16.2%	
Monthly or less	28.5%	33.6%		3–4	27.5%	35.3%		Less than monthly	42.7%	57.3%	
2–4 times a month	48.2%	40.9%		5–6	36.9%	20.8%		Monthly	41.4%	23.9%	
2–3 times a week	15.0%	14.3%		7–9	18.8%	8.2%		Weekly	6.1%	2.5%	
Four times a week or more	1.1%	1.8%		10 or more	4.7%	2.2%		Daily or almost daily	< 1%	< 1%	
**Females**											
Frequency of alcohol consumption			<0.001	Usual consumption^a^			<0.001	Binge drinking (6 units or more)			<0.001
Never	7.4	9.8		1–2	12.0	35.3		Never	10.9%	18.5%	
Monthly or less	30.4	36.0		3–4	30.4	37.3		Less than monthly	45.5%	59.5%	
2–4 times a month	48.5	40.3		5–6	40.0	20.3		Monthly	39.2%	20.5%	
2–3 times a week	13.1	12.5		7–9	15.2	6.2		Weekly	4.3%	1.5%	
Four times a week or more	0.7	1.3		10 or more	2.5	0.9		Daily or almost daily	< 1%	< 1%	
**Males**											
Frequency of alcohol consumption			<0.001	Usual consumption^a^			<0.001	Binge drinking (6 units or more)			<0.001
Never	7.0	8.2		1–2	12.4	29.1		Never	6.6%	10.4%	
Monthly or less	23.5	27.4		3–4	20.2	30.0		Less than monthly	35.3%	51.7%	
2–4 times a month	47.3	42.5		5–6	28.9	22.2		Monthly	47.1%	32.6%	
2–3 times a week	20.0	19.1		7–9	28.1	13.2		Weekly	10.8%	5.1%	
Four times a week or more	2.3	2.9		10 or more	10.5	5.4		Daily or almost daily	< 1%	< 1%	

*Proportions for usual consumption excludes those who answered “never” to frequency of alcohol consumption*.

a *Refers to standard drinks*.

### Changes Related to Age Groups

**Figure 2:**
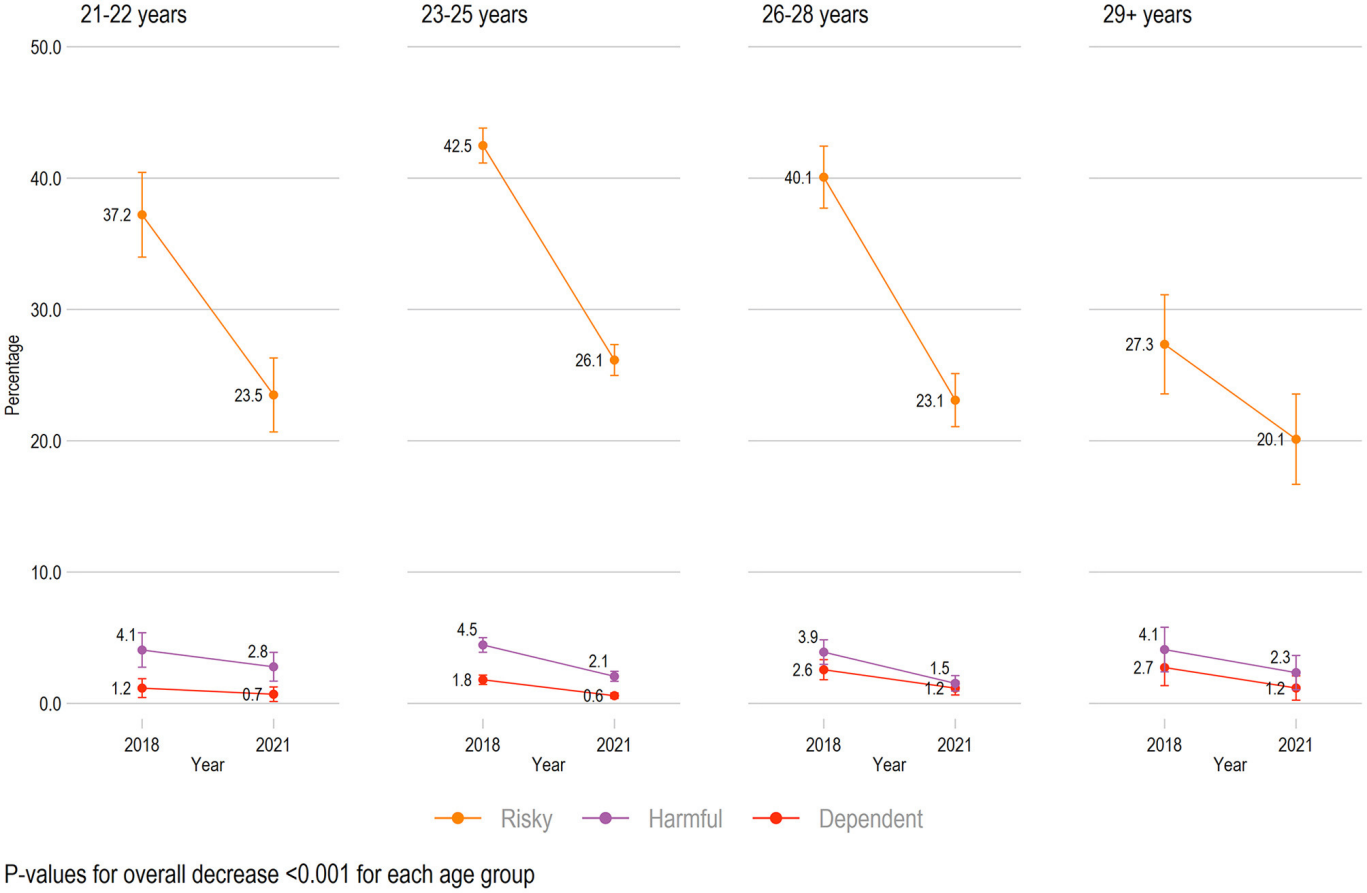
Trends in alcohol use across age groups from 2018 to 2021 (*n* = 8,287). All rates are adjusted by gender and geographical location. Scores are based on Alcohol Use Disorder Identification Test: Risky = range 8–15, Harmful = range 15–19, Dependent = range 20–40. Rates of “No or low risk alcohol use” are not shown.

We found a significant decrease in the mean alcohol problems scores (total AUDIT score) across all age groups from 2018 to 2021 (all *ps* < 0.001, Supplementary Table A2). The rates of risky alcohol use decreased across all age groups (all *ps* < 0.001, Figure 2), while the rates of harmful alcohol use decreased for the age groups 23–25 years (*p* < 0.001) and 26–28 (*p* < 0.001) but not for the 21–22 (*p* = 0.144) and 29+ years age group (*p* = 0.122). The rates of dependent alcohol use decreased for all age groups (all *ps* < 0.01) except those aged 21–22 years (*p* = 0.424) and 29+ years (*p* = 0.077).

### Changes Related to Geographical Study Locations

**Figure 3:**
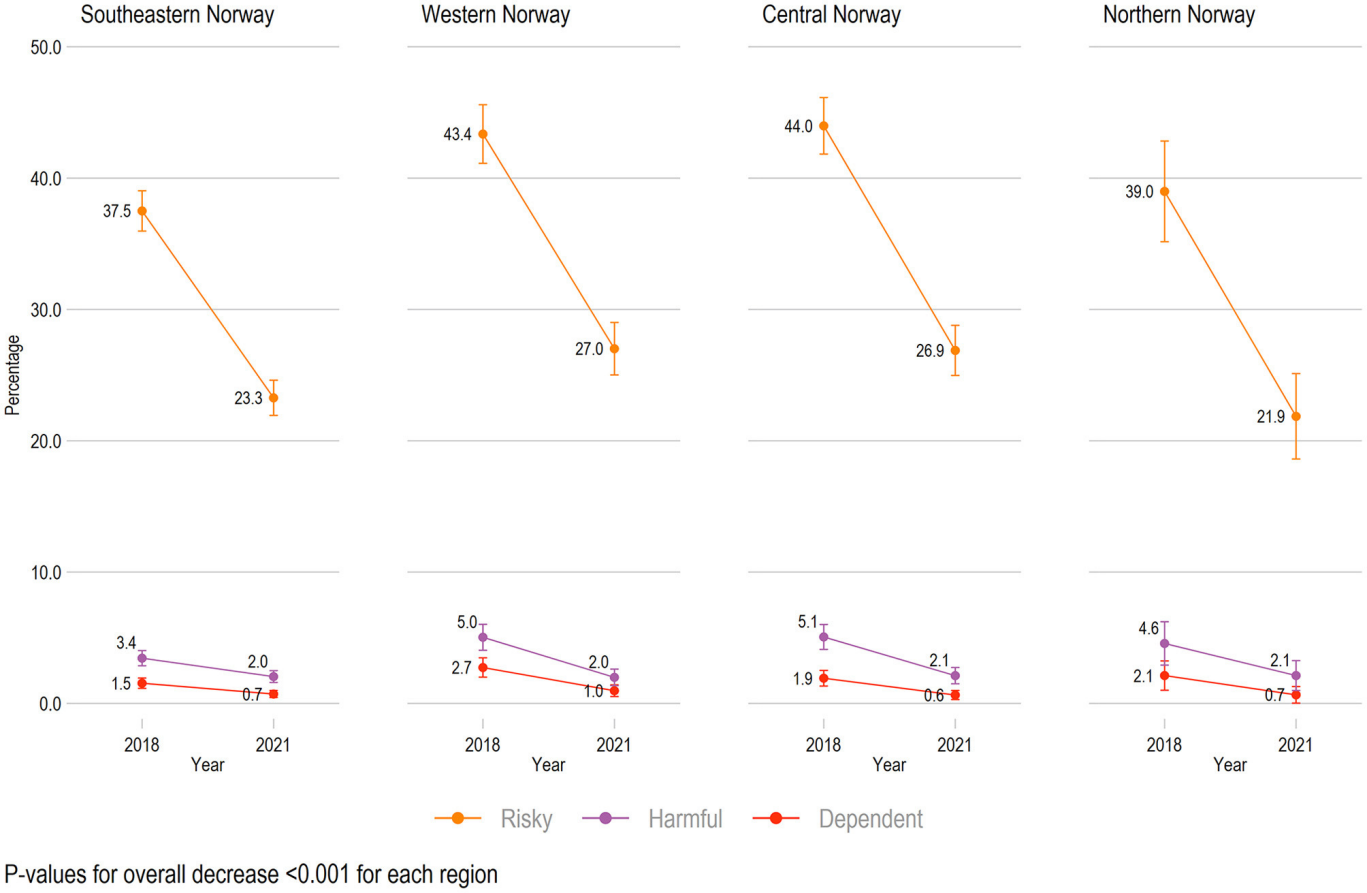
Trends in alcohol use across geographical study locations from 2018 to 2021 (*n* = 8,287). All rates are adjusted by age and gender. Scores are based on Alcohol Use Disorder Identification Test: Risky = range 8-15, Harmful = range 15-19, Dependent = range 20–40. Rates of “No or low risk alcohol use” are not shown.

We also found a significant decrease in the mean alcohol problems scores (total AUDIT score) across all geographical study locations from 2018 to 2021 (all *ps* < 0.001, Supplementary Table A3). The rates of risky, harmful, and dependent alcohol use decreased significantly across all regions (all *ps* < 0.05, Figure 3).

### Changes Across Categories of Alcohol Use

**Figure 4:**
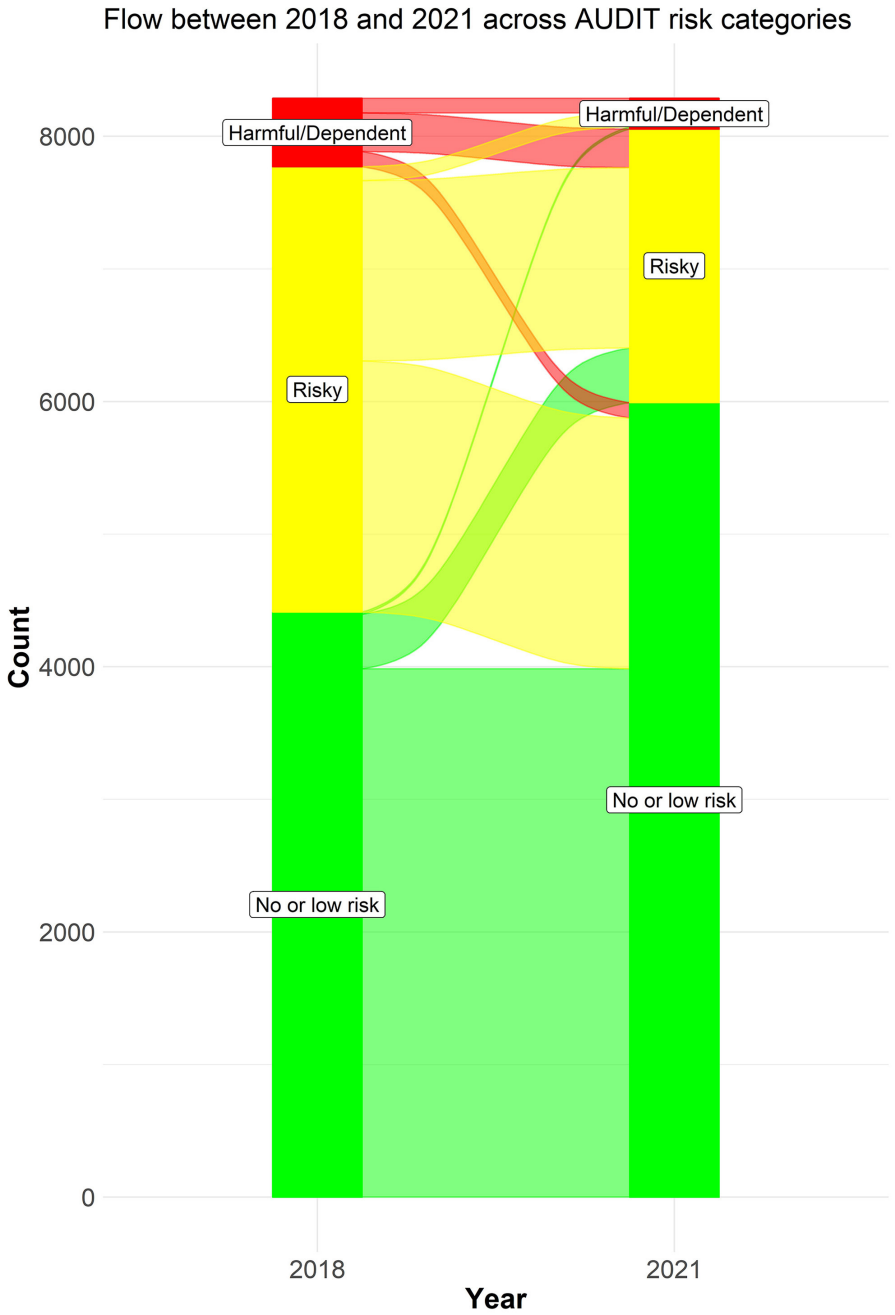
Flows from 2018 to 2021 for AUDIT-categories (*n* = 8,287). Scores are based on Alcohol Use Disorder Identification Test: No or low risk = 0–7, Risky = range 8–15, Harmful/Dependent = range 15–40, Dependent = range 20–40.

The alluvial plot (Figure 4) indicates the number of flows identified between 2018 and 2021 across AUDIT-categories. Overall, 27.7% of the participants had a downward flow, compared to 6.5% with an upward flow, while 65.8% had a stable flow (Supplementary Table A4). The most common flow was the stable no or low risk flow (48.1), while the most common changing flow was for risky to no or low risk flow (22.8%).

## Discussion

The present study is the first to assess change in alcohol use and alcohol-related problems among Norwegian higher education students during the COVID-19 pandemic. Using a longitudinal dataset from two cross-sectional national surveys, we were able to compare pre-pandemic levels of alcohol-related problems from 2018 with comparable data from March 2021, during the lockdown of student campuses. The main finding from the present study was a sharp decline in alcohol-related problems among Norwegian students in higher education during this period across age-, gender-, and geographical strata.

The present study adds novel results as no previous studies have evaluated alcohol use among Norwegian students in higher education during the pandemic. Our results are nevertheless consistent with other findings from the Norwegian general populations, that also has reported declines in alcohol use during this period (12, 31). The reduction of alcohol use in the general population has primarily been driven by a reduction in heavy episodic drinking (12). In comparison, the present study reported significant decreased rates of both risky, harmful, and dependent alcohol use. Of note, one previous study reported that little change in alcohol consumption was seen in Norway (32), something that was not supported in the present study. In a previous cross-sectional study of self-reported change in alcohol consumption during first 6 weeks of national lock-down found that 13% reported increased alcohol consumption, and that this increase was more commonly reported by people with economic worries, in quarantine and those studying/working from home (33).

Notwithstanding, our results support a range of international studies that have reported a significant reduction in alcohol use among students in higher education throughout the pandemic (14–18). On the other hand, two previous studies reported that alcohol use among US university and college students, respectively, increased significantly immediately after the COVID-19 related campus/college closure during March 2020, compared with the weeks prior to closure (19, 20). Elevated psychological distress was associated with steeper increases in alcohol use (19). A potential explanation of this increase, compared with the decreases in alcohol use found in other studies, may be related to the timing of the measurement of alcohol use. In the immediate phase of the pandemic, it is possible that pandemic-related fears have been stronger and more acute than during later stages of the pandemic, when a larger degree of habituation to the situation is possible. The present study used data on student alcohol use collected from March to April 2021, in other words after ~ 1 year into the pandemic. Thus, it is possible that changes in student alcohol use immediately after the lockdown were different in both direction and magnitude compared with the data employed in the present study.

Interestingly, the decline in alcohol-related problems among Norwegian students in the present study was generally observed across gender, age groups, and geographical locations. Although rates of students with harmful and dependent alcohol use did not significantly change from 2018 to 2021 in the youngest and oldest age groups in our sample (i.e., 21–22 years of age, and 29+ years of age), we observed reductions also in these groups that were similar in magnitude to the other age groups. Hence, the lack of statistically significant change in harmful and dependent alcohol use in the youngest and oldest age group is likely a result of low power in these analyses, as these age groups were the smallest in our sample. Overall, our findings strongly suggests that the decline in alcohol use was general in the student population and not restricted to specific groups or certain areas of Norway. Although the present study did not address potential mechanisms behind the observed reduction in the student population, we suggest that our findings are likely explained by reduced socialization, less parties and limited access to licensed premises such as bars and night clubs due to the social distancing measures that were implemented throughout the country during this period. This interpretation is consistent with a previous report (14). Our findings may further be interpreted to indicate that students' alcohol use to a large extent is related to social contexts.

The findings of the present study differ from a recent study from eight European countries, where it was reported that among people with an initial high level of alcohol consumption, alcohol use increased during the first months of the COVID-19 pandemic (21). Students are a high-risk drinking population, due to a large proportion of individuals with high or very high levels of alcohol use and frequent binge drinking (1, 2). However, in contrast to such high-risk drinkers in the general population, it is interesting to note that students in general decreased rather than increased their drinking. Thus, our findings may suggest that students as a high-risk drinking group is somewhat different from high-risk drinkers in the adult general population. On the other hand, our results suggest that most students remained stable within his/her original AUDIT-risk category from 2018 to 2021 (e.g., scoring at “risky alcohol use” in both surveys). However, 28% of the students fell one category from 2018 to 2021, indicating a reduction of alcohol use and alcohol-related problems, while 6.5% rose one category in the same period, indicating an increase in alcohol use and alcohol-related problems during this period. Thus, although the data from the present study clearly points toward a general decline of alcohol-related problems in the student population, there may be vulnerable students or sub-groups that have developed alcohol-related problems during the pandemic, and they need to be identified and offered adequate help.

An important implication of the present study relates to what could be expected when pandemic conditions eventually change, and student conditions turn back to normal. It is well-established that high levels of alcohol use are associated with negative outcomes. We find it unlikely that the observed decrease in alcohol use and alcohol-related problems among the students are sustained for any period of time after social distancing measures are removed, and a focus on universal preventive initiatives to reduce future student alcohol use are thus encouraged. For example, Norwegian students have repeatedly called for more alcohol-free social arrangements (34).

### Strengths and Limitations

The main strength of the present study was that baseline data from 2018 on students' alcohol-related problems was available prior to the survey conducted during the COVID-19 pandemic, as well as the follow-up measure from 2021, during a time when wide-ranging restrictions related to the pandemic were in place. The linked longitudinal study sample was sufficiently large to carry out sub-group analyses of trends in alcohol-related problems. Also, the inclusion of a detailed and validated instrument for assessing potential alcohol-related problems (AUDIT) adds as a strength.

An important limitation of the present study was modest response rate of the two SHoT studies. It is possible that the web-based approach may have contributed to a low response rate, as paper-based surveys and face-to-face interviews typically yield better overall response rates compared with approaches using electronical platforms (35). Another limitation was the 72% female composition of the sample, which is somewhat higher that the true ≈60% female composition among students in higher education in Norway (36). In addition, the age composition of our sample had an overweight of students aged 23–25 years. There were also slight differences between students of the included sample (i.e., students that participated in both surveys) compared with those that only participated in SHoT2018, including that they were more likely to be female and younger. We cannot rule out that this overrepresentation of female students and relatively young students may have contributed to some bias in our results. However, rates of alcohol-related problems were adjusted by gender and age, contributing to counteract this limitation. Also, all the stratified analyses yielded similar results, namely a decline in alcohol-related problems.

Also, while alcohol use in the student population have seen a sharp decline from 2018 to 2021, it is still possible that vulnerable groups (e.g., students that have experienced high levels of psychological distress during the pandemic) have had increases in alcohol use and alcohol-related problems during this period. Moderation analyses could have shed light on this possibility. This was, however, beyond the scope of the present study. Finally, some students may have dropped out from their studies during the COVID-19 pandemic, in part due to difficulties with home-studying. As it is likely that many of these students did not participate in the second SHoT-survey, our result may not be fully generalized to this sub-group of former students.

## Conclusions

Alcohol use is an important health concern among Norwegian college and university students, largely due to high volume of alcohol use in student social settings. The present study clearly demonstrates a reduction in alcohol-related problems among students during the COVID-19 pandemic, which is likely explained by reduced availability to the ordinary student social areas. Universal preventive measures are needed to limit increase in alcohol use among students when restrictions related to the pandemic are lifted.

## Data Availability Statement

The datasets presented in this article are not readily available because of privacy regulations from the Norwegian Regional Committees for Medical and Health Research Ethics (REC). Approval from REC (https://helseforskning.etikkom.no) is a pre-requirement. Guidelines for access to SHoT data are found at: https://www.fhi.no/en/more/access-to-data. Requests to access the datasets should be directed to borge.sivertsen@fhi.no.

## Ethics Statement

The studies involving human participants were reviewed and approved by the Regional Committee for Medical and Health Research Ethics in Western Norway. The patients/participants provided their written informed consent to participate in this study.

## Author Contributions

OH, BS, and JS contributed to conception and design of the study. BS prepared the dataset. OH and JS performed the statistical analysis. OH wrote the first draft of the manuscript. JS wrote sections of the manuscript and created the figures. All authors contributed to the manuscript revision, read, and approved the submitted version.

## Funding

SHoT2018 has received funding from the Norwegian Ministry of Education and Research (2017) and the Norwegian Ministry of Health and Care Services (2016).

## Conflict of Interest

The authors declare that the research was conducted in the absence of any commercial or financial relationships that could be construed as a potential conflict of interest.

## Publisher's Note

All claims expressed in this article are solely those of the authors and do not necessarily represent those of their affiliated organizations, or those of the publisher, the editors and the reviewers. Any product that may be evaluated in this article, or claim that may be made by its manufacturer, is not guaranteed or endorsed by the publisher.
