# Supplementary material for: The Extent of Alcohol-Related Problems Among College and University Students in Norway Prior to and During the COVID-19 Pandemic
Source: Front Public Health. 2022 May 26;10:876841. doi: 10.3389/fpubh.2022.876841 (PMC9204355; doi:10.3389/fpubh.2022.876841)
Supplement: Supplementary file 1 [file Table_1.DOCX]

**Additional tables**

**Table A1. Trends in AUDIT-scores across gender from 2018 to 2021 (n=8,287)**

| Gender | 2018  (CI95%) | 2021  (CI95%) |
| --- | --- | --- |
| **Women** |  |  |
| Mean | 7.2  (7.1-7.3) | 5.3  (5.2-5.4) |
| No or low risk alcohol use (AUDIT-range: 0-7), % | 56.4  (55.1-57.6) | 75.8  (74.8-76.9) |
| Risky alcohol use (AUDIT-range: 8-15), % | 38.6  (37.4-39.9) | 22.1  (21.1-23.2) |
| Harmful alcohol use (AUDIT-range: 16-19), % | 3.6  (3.2-4.1) | 1.6  (1.3-1.9) |
| Dependent alcohol use (AUDIT-range: 20-40), % | 1.4  (1.1-1.7) | 0.5  (0.3-0.7) |
| **Men** |  |  |
| Mean | 8.5  (8.3-8.7) | 6.7  (6.5-6.8) |
| No or low risk alcohol use (AUDIT-range: 0-7), % | 45.0  (43.0-47.0) | 63.0  (61.0-64.9) |
| Risky alcohol use (AUDIT-range: 8-15), % | 45.5  (43.5-47.6) | 32.3  (30.4-34.2) |
| Harmful alcohol use (AUDIT-range: 16-19), % | 6.0  (5.1-7.1) | 3.2  (2.6-4.1) |
| Dependent alcohol use (AUDIT-range: 20-40), % | 3.5  (2.8-4.3) | 1.5  (1.1-2.1) |
|  |  |  |

*Notes*: Crude means and proportions; AUDIT = Alcohol Use Disorders Identification Test; CI = confidence interval.

**Table A2. Trends in AUDIT-scores across age groups from 2018 to 2021 (n=8,287).**

| Age groups | 21-22 years  (CI95%) | 23-25 years  (CI95%) | 26-28 years  (CI95%) | 29+ years  (CI95%) |
| --- | --- | --- | --- | --- |
| **2018** |  |  |  |  |
| Mean | 7.0  (6.7-7.3) | 7.7  (7.6-7.9) | 7.6  (7.4-7.8) | 6.5  (6.1-7.0) |
| No or low risk alcohol use (AUDIT-range: 0-7), % | 57.6  (54.2-60.8) | 51.3  (49.9-52.6) | 53.5  (51.0-55.9) | 65.8  (61.6-69.8) |
| Risky alcohol use (AUDIT-range: 8-15), % | 37.2  (34.0-40.5) | 42.5  (41.2-43.8) | 40.1  (37.7-42.5) | 27.3  (23.6-31.4) |
| Harmful alcohol use (AUDIT-range: 16-19), % | 4.1  (2.9-5.6) | 4.5  (3.9-5.0) | 3.9  (3.1-5.0) | 4.1  (2.7-6.2) |
| Dependent alcohol use (AUDIT-range: 20-40), % | 1.2  (0.6-2.1) | 1.8  (1.5-2.2) | 2.6  (1.9-3.5) | 2.7  (1.6-4.6) |
| **2021** |  |  |  |  |
| Mean | 5.7  (5.5-6.0) | 5.8  (5.7-5.9) | 5.6  (5.4-5.8) | 5.1  (4.7-5.5) |
| No or low risk alcohol use (AUDIT-range: 0-7), % | 73.0  (70.0-75.9) | 71.2  (70.0-72.4) | 74.2  (72.0-76.3) | 76.4  (72.5-79.9) |
| Risky alcohol use (AUDIT-range: 8-15), % | 23.5  (20.8-26.4) | 26.1  (25.0-27.3) | 23.1  (21.1-25.2) | 20.1  (16.9-23.8) |
| Harmful alcohol use (AUDIT-range: 16-19), % | 2.8  (1.9-4.1) | 2.1  (1.7-2.5) | 1.5  (1.0-2.3) | 2.3  (1.3-4.1) |
| Dependent alcohol use (AUDIT-range: 20-40), % | 0.7  (0.3-1.5) | 0.6  (0.4-0.8) | 1.2  (0.7-1.8) | 1.2  (0.5-2.6) |
|  |  |  |  |  |

*Notes*: Crude means and proportions; AUDIT = Alcohol Use Disorders Identification Test; CI = confidence interval.

**Table A3. Trends in AUDIT-scores across study location from 2018 to 2021 (n=8,287).**

| Geographical locations | Southeastern Norway (CI95%) | Western Norway  (CI95%) | Central Norway  (CI95%) | Northern Norway  (CI95%) |
| --- | --- | --- | --- | --- |
| **2018** |  |  |  |  |
| Mean | 7.0  (6.9-7.2) | 8.1  (7.9-8.4) | 8.0  (7.8-8.2) | 7.6  (7.3-8.0) |
| No or low risk alcohol use (AUDIT-range: 0-7), % | 57.5  (55.9-59.1) | 48.9  (46.6-51.1) | 49.0  (46.9-51.2) | 54.3  (50.3-58.2) |
| Risky alcohol use (AUDIT-range: 8-15), % | 37.5  (36.0-39.1) | 43.4  (41.1-45.6) | 44.0  (41.8-46.1) | 39.0  (35.2-42.9) |
| Harmful alcohol use (AUDIT-range: 16-19), % | 3.4  (2.9-4.1) | 5.0  (4.1-6.1) | 5.1  (4.2-6.1) | 4.6  (3.2-6.5) |
| Dependent alcohol use (AUDIT-range: 20-40), % | 1.5  (1.2-2.0) | 2.7  (2.1-3.6) | 1.9  (1.4-2.6) | 2.1  (1.2-3.6) |
| **2021** |  |  |  |  |
| Mean | 5.4  (5.3-5.6) | 6.0  (5.8-6.2) | 5.9  (5.7-6.1) | 5.5  (5.2-5.9) |
| No or low risk alcohol use (AUDIT-range: 0-7), % | 74.0  (72.5-75.3) | 70.0  (67.9-72.1) | 70.4  (68.3-72.3) | 75.4  (71.8-78.6) |
| Risky alcohol use (AUDIT-range: 8-15), % | 23.3  (21.9-24.6) | 27.0  (25.0-29.1) | 26.9  (25.0-28.9) | 21.9  (18.8-25.3) |
| Harmful alcohol use (AUDIT-range: 16-19), % | 2.0  (1.6-2.5) | 2.0  (1.4-2.7) | 2.1  (1.6-2.8) | 2.1  (1.2-3.6) |
| Dependent alcohol use (AUDIT-range: 20-40), % | 0.7  (0.5-1.0) | 1.0  (0.6-1.5) | 0.6  (0.4-1.1) | 0.7  (0.2-1.7) |
|  |  |  |  |  |

*Notes*: Crude means and proportions; AUDIT = Alcohol Use Disorders Identification Test; CI = confidence interval.

**Table A4. Flows from 2018 to 2021 for AUDIT-categories (n=8,287)**

| **Flows** | **% (CI95%)** |
| --- | --- |
| Downward flows |  |
| Risky to no or low risk flow | 22.8 (22.0-23.8) |
| Harmful/dependent to risky flow | 3.5 (3.1-3.9) |
| Harmful/dependent to no or low risk flow | 1.4 (1.2-1.7) |
| *Total* | *27.7* |
| Upward flows |  |
| No or low risk to risky flow | 5.0 (4.5-5.5) |
| Risky to harmful/dependent flow | 1.3 (1.0-1.5) |
| No or low risk to harmful/dependent flow | 0.2 (0.1-0.3) |
| *Total* | *6.5* |
| Stable flows |  |
| Stable no or low risk flow | 48.1 (47.0-49.2) |
| Stable risky flow | 16.4 (15.6-17.2) |
| Stable harmful/dependent flow | 1.3 (1.1-1.6) |
| *Total* | *65.8* |
|  |  |

*Notes*: AUDIT = Alcohol Use Disorders Identification Test; CI = confidence interval.

Scores are based on Alcohol Use Disorder Identification Test: No or low risk = 0-7; Risky = range 8-15; Harmful/Dependent = range 15-40; Dependent = range 20-40.
